# Evolutionary Plasticity of Habenular Asymmetry with a Conserved Efferent Connectivity Pattern

**DOI:** 10.1371/journal.pone.0035329

**Published:** 2012-04-13

**Authors:** Aldo Villalón, Mauricio Sepúlveda, Néstor Guerrero, Margarita M. Meynard, Karina Palma, Miguel L. Concha

**Affiliations:** 1 Biomedical Neuroscience Institute (BNI), Santiago, Chile; 2 Anatomy and Developmental Biology Program, Faculty of Medicine, Institute of Biomedical Sciences, Universidad de Chile, Santiago, Chile; 3 Faculty of Medicine, Universidad Diego Portales, Santiago, Chile; Harvard University, United States of America

## Abstract

The vertebrate habenulae (Hb) is an evolutionary conserved dorsal diencephalic nuclear complex that relays information from limbic and striatal forebrain regions to the ventral midbrain. One key feature of this bilateral nucleus is the presence of left-right differences in size, cytoarchitecture, connectivity, neurochemistry and/or gene expression. In teleosts, habenular asymmetry has been associated with preferential innervation of left-right habenular efferents into dorso-ventral domains of the midbrain interpeduncular nucleus (IPN). However, the degree of conservation of this trait and its relation to the structural asymmetries of the Hb are currently unknown. To address these questions, we performed the first systematic comparative analysis of structural and connectional asymmetries of the Hb in teleosts. We found striking inter-species variability in the overall shape and cytoarchitecture of the Hb, and in the frequency, strength and to a lesser degree, laterality of habenular volume at the population level. Directional asymmetry of the Hb was either to the left in D. rerio, E. bicolor, O. latipes, P. reticulata, B. splendens, or to the right in F. gardneri females. In contrast, asymmetry was absent in P. scalare and F. gardneri males at the population level, although in these species the Hb displayed volumetric asymmetries at the individual level. Inter-species variability was more pronounced across orders than within a single order, and coexisted with an overall conserved laterotopic representation of left-right habenular efferents into dorso-ventral domains of the IPN. These results suggest that the circuit design involving the Hb of teleosts promotes structural flexibility depending on developmental, cognitive and/or behavioural pressures, without affecting the main midbrain connectivity output, thus unveiling a key conserved role of this connectivity trait in the function of the circuit. We propose that ontogenic plasticity in habenular morphogenesis underlies the observed inter-species variations in habenular asymmetric morphology.

## Introduction

The vertebrate habenulae (Hb) is an evolutionary conserved dorsal diencephalic nuclear complex that relays information from limbic and striatal forebrain regions to the ventral midbrain and hindbrain, and has been involved in a wide range of cerebral functions linked to the modulation of monoaminergic neurons [1], [2]. One key feature of this nuclear complex that becomes conspicuous in anamniotes, is the presence of left-right differences in size, cytoarchitecture, connectivity, neurochemistry and/or gene expression [3]. Despite the conservation of left-right asymmetry as a habenular trait per se, the type, strength and laterality (left or right directionality) of asymmetry exhibit considerable variation among species of different vertebrate groups [3]. For example, in the sea lamprey Petromyzon marinus the right Hb is considerably larger and more stratified than the left Hb [4] while in the frog Rana esculenta the left Hb has a unique lateral subnucleus that is not observed in the right Hb [5]. In amniotes, habenular asymmetry is prominent only in a few species of lizards such as Uta stansburiana [6] but in most cases it involves only subtle volumetric differences between left and right habenular nuclei [7], [8], [9]. In teleosts, scattered reports of qualitative nature have shown that the Hb of different species can be either symmetric or enlarged on one side (left or right) [3] and those discrepancies seem to extend to individuals within a single species i.e. Geophagus brasiliensis [10]. Although structural asymmetries of the Hb appear remarkably variable among teleosts, systematic inter-species comparisons using quantitative approaches are needed to corroborate the accuracy of these observations. An additional feature of the teleost Hb, which is not observed in species of other vertebrates groups (e.g. salamanders, frog and mice) [11], is the target selectivity that left and right habenular projection neurons show for dorsal and ventral domains of the midbrain interpeduncular nucleus (IPN), respectively [11], [12], [13]. The degree of conservation of such “laterotopic” habenular innervation of the IPN, and its relation to the observed variations in structural asymmetry of the Hb remain unclear. To address these questions we performed the first systematic comparative analysis of habenular asymmetric morphology and left-right Hb-IPN connectivity, using seven species of fresh-water teleosts showing relatively similar size at adult stages: Danio rerio and Epalzeorhynchos bicolor (Order Cypriniformes); Oryzias latipes (Order Beloniformes); Poecilia reticulata and Fundulopanchax gardneri (Order Cyprinodontiformes); Pterophyllum scalare and Betta splendens (Order Perciformes). We found striking variability in the presence, strength and to a lesser extent, laterality of population level asymmetry in the Hb, which co-existed with an overall conservation of the laterotopic pattern of Hb-IPN connectivity. These results are consistent with a sequential modular organisation in the ontogeny of habenular asymmetry and suggest that the developmental mechanisms underlying asymmetric morphogenesis promote structural flexibility in the Hb without largely affecting the main left-right efferent connectivity outcome.

## Results

### The overall shape and cytoarchitecture of the teleost habenulae exhibit large inter-species variation

**Figure 1:**
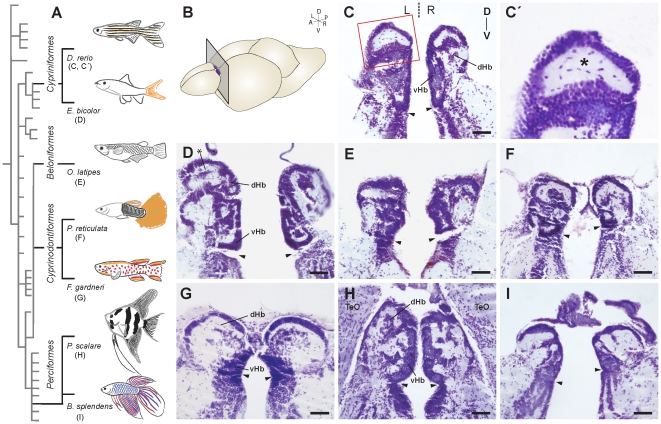
Cytoarchitectonic organisation of the habenulae in teleosts. (A) Drawings of adult male individuals belonging to different teleost species, placed in the context of a cladogram of the teleost lineage according to Nelson [28]. (B) Schematic representation of a teleost brain (e.g. D rerio), showing the location and orientation of histological sections shown in C–I. (C–I) Photomicrographs of cresyl-violet stained 10 µm-thick coronal sections taken at a midpoint between rostral and caudal ends of the Hb as shown in B. Each panel corresponds to a single species, as indicated in the letter code of the left diagram. C′ is a magnification of the square region depicted in C. Dorsal is to the top, and left is to the left. Arrowheads point to the subhabenular sulcus. Asterisks indicate the position of the dorsal-most neuropil region of the dorsal habenulae that is surrounded by a shell of cell bodies in some species. Abbreviations: A (anterior), D (dorsal), dHb (dorsal Hb), L (left), P (posterior), R (right), TeO (Optic Tectum), V (ventral), vHb (ventral Hb). Scale bars: 50 µm.

**Figure 2:**
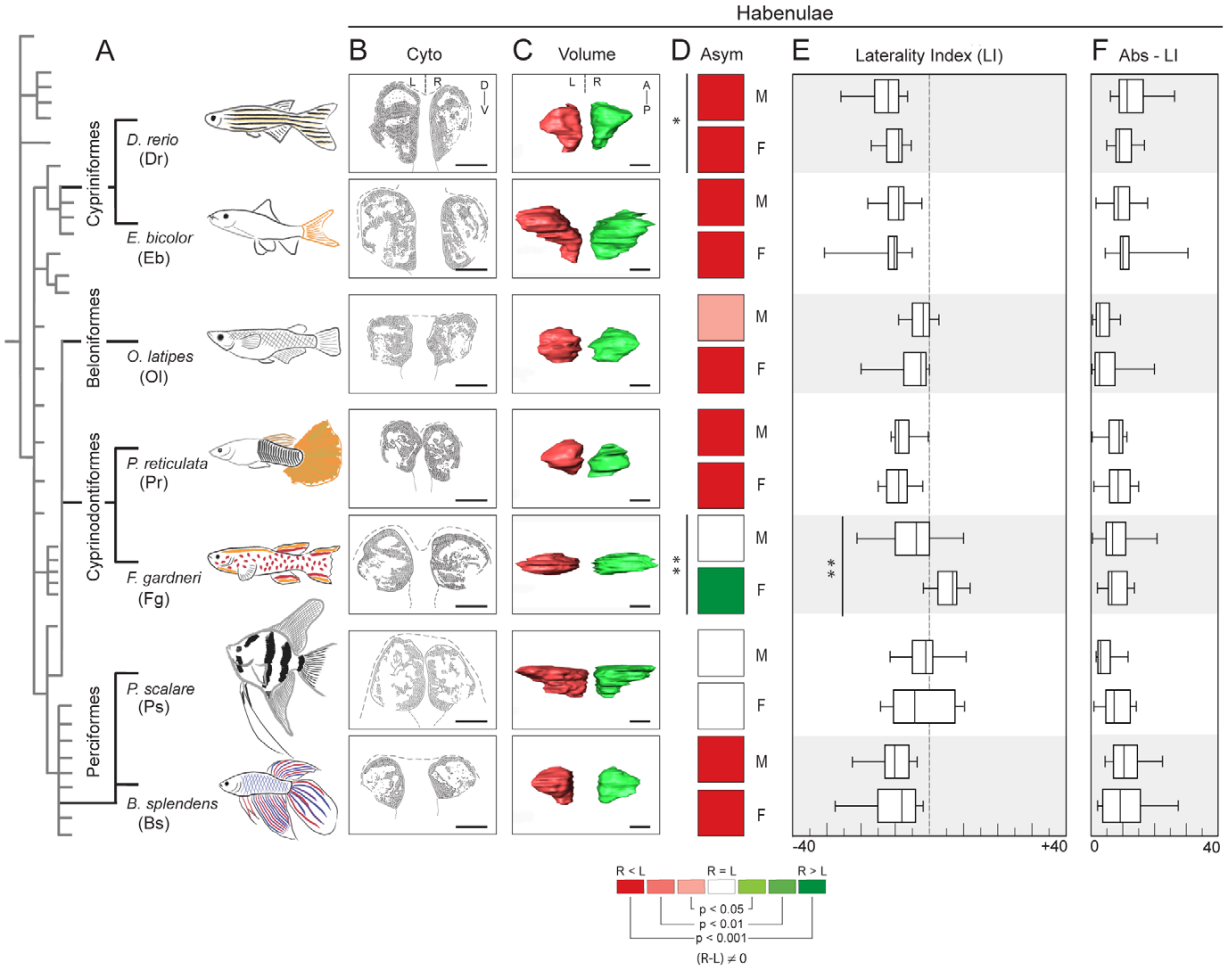
Comparative analysis of volumetric asymmetry in the habenulae of teleosts. (A) Drawings of adult male individuals belonging to different teleost species, placed in the context of a cladogram of the teleost lineage according to Nelson [28]. For each species, the corresponding panels from columns B to F are aligned horizontally. (B) Schematics of habenular cytoarchitecture obtained from the cresyl-violet stained coronal sections of the Hb shown in Figure 1. (C) Volumetric models of the Hb as seen dorsally, with anterior to the top. Volumes of the left and right Hb have been differentially coloured in red and green, respectively. (D) Colour code diagram indicating the presence of statistically significant laterality of habenular volume at the population level ([R-L]≠0). For each box, the presence of left- or right- directional asymmetries, and the corresponding p values, have been coloured according to the colour scale given below. Vertical lines and asterisks placed on the left side of some pairs of boxes indicate sex-specific significant differences in habenular asymmetry (* = p<0.05; ** = p<0.01). (E) Box plots indicating the scores of habenular Laterality Index for each species and sex. Positive and negative values indicate right- and a left- sided laterality of habenular volume, respectively. Values reveal the strength and directionality of habenular asymmetry at the population level. The vertical line with asterisks placed indicates sex-specific significant differences in Laterality Index (** = p<0.01). (F) Box plots indicating the absolute values of Laterality Index (abs-LI) for each species and sex, which reveal the strength of habenular asymmetry at the individual level. For each plot shown in E and F, the box depicts the interquartile range containing 50% of the data around the median (vertical line inside the box), and the whisker depicts maximum and minimum vales. Abbreviations: A (anterior), Asym (asymmetry), Cyto (cytoarchitecture), D (dorsal), F (females), L (left), M (males), P (posterior), R (right), V (ventral). Scale bar: 100 µm for column B, and 200 µm for column C.

Analysis of cresyl-violet stained 10 µm-thick coronal sections of the Hb (Fig. 1) and of three-dimensional models generated after segmenting the borders of the Hb in its entire rostro-caudal extent (Fig. 2C) revealed a striking variability in the overall shape and cytoarchitecture of the Hb among the species of teleosts examined. Interestingly, inter-species variability was more pronounced across orders than within a single order. For example, the Hb of D. rerio and E. bicolor (Order Cypriniformes) were elongated along the rostro-caudal axis, with most of the volume allocated to rostral positions of the structure (Fig. 2C). In coronal sections, the Hb showed an isosceles triangle-like shape, elongated in the dorso-ventral axis, and with the shorter side facing dorsal (Fig. 1C,D). The border between dorsal (dHb) and ventral (vHb) habenular nuclei was easily distinguishable on the basis of pure cytoarchitecture as previously shown for zebrafish [14]. The vHb occupied a ventro-medial position and contained cells slightly smaller, more densely packed, and with more intense staining than cells of the dHb (Fig. 1C,D). In the dHb, a shell of cell bodies surrounded the dorsal-most neuropil region on both sides, although this feature was more notorious on the larger left-sided dHb (asterisks in Fig. 1C′,D). In contrast to Cypriniformes, the Hb in species of Beloniformes and Cyprinodontiformes showed spherical (O. latipes), oval (P. reticulata) or dish-like (F. gardneri) shapes when observed in dorsal views of rostro-caudal volumetric projections (Fig. 2C). In coronal sections, the Hb acquired a quadrangle-to-round shape with a complex cytoarchitectonic arrangement of cellular and neuropil regions, in which the boundary between the dHb and vHb was not clearly distinguishable, with the exception of F. gardneri (Fig. 1E–G). In Perciformes (P. scalare, B. splendens), the Hb was elongated in the rostro-caudal direction with a predominant rostral volume allocation (Fig. 2C) as seen in Cypriniformes, and coronal sections revealed a quadrangle-to-round shape (Fig. 1H–I) similar to the Hb of Cyprinodontiformes. In P. scalare, the cytoarchitectonic organisation allowed a broad distinction of dHb and vHb.

### The frequency, strength and laterality of habenular asymmetry varies among teleosts

We examined the extent of volumetric asymmetries in the Hb using the three-dimensional models explained above (Fig. 2C). An overall comparative perspective of the statistically significant differences between right and left habenular volumes ([R-L]≠0) revealed substantial variability in the frequency of asymmetry among the examined teleost species (Fig. 2D and Table 1). As observed for habenular shape and cytoarchitecture, inter-species variations in habenular asymmetry were also more frequent across teleost orders than within a single order. In the great majority of species showing significant asymmetry at the population level, the volume of the left Hb was enlarged compared to the right Hb (Fig. 2D, red boxes) with the sole exception of F. gardneri females, in which the right Hb was larger than the left counterpart (Fig. 2D, green box). Left-sided directional asymmetry was specially prominent in both species of Cypriniformes (D. rerio, E. bicolor), B. splendens (Perciformes) and P. reticulata (Cyprinodontiformes), as revealed by the highest negative values of habenular Laterality Index (LI) among the examined species (LI*_Dr_*_-male_ = −11.6; LI*_Dr_*_-female_ = −9.11; LI*_Eb_*_-male_ = −8.73; LI*_Eb_*_-female_ = −10.7; LI*_Bs_*_-male_ = −10.2; LI*_Bs_*_-female_ = −8.25; LI*_Pr_*_-male_ = −9.11; LI*_Pr_*_-female_ = −8.95; Median) (Fig. 2E and Table 1). In females of F. gardneri (Cyprinodontiformes), directional asymmetry of the Hb was also strong but directed to the right side (LI*_Fg_*_-female_ = 6.52). In contrast, directional asymmetry of habenular volume was rather weak and directed to the left in O. latipes (Beloniformes) (LI*_Ol_*_-male_ = −2.23; LI*_Ol_*_-female_ = −2.58), and absent in P. scalare (Perciformes) and males of F. gardneri (Cyprinodontiformes) (Fig. 2D, white boxes, and Table 1). For the latter group, lack of population level asymmetry was associated with a large dispersion of LI values across the zero reference (see size of the interquartile range and max/min values in box plots of Fig. 2E and Table 1). Indeed, evaluation of the absolute scores of habenular asymmetry ([abs(R-L)]≠0) showed that all teleost species lacking population level asymmetry did exhibit significant asymmetry at the individual level (P. scalare males p = 0.0069, and females p = 0.00029; F. gardneri males p = 0.01063). In addition, the absolute scores of LI (abs-LI) revealed that habenular asymmetries in these species were particularly strong at the individual level (abs-LI*_Fg_*_-male_ = 6.66; abs-LI*_Ps_*_-female_ = 8.13) (Fig. 2F and Table 1).

**Table 1:** Asymmetry and laterality index of the habenulae in teleosts.

		Asymmetry [R-L]≠0			Laterality Index					
					Directional			Absolute		
	Sex	Median (mm^3^)	Min/Max (mm^3^)	*p*-Value	Median	Min/Max	*p*-Value	Median	Min/Max	*p*-Value
***D. rerio***	Male	−0.00059	−0.00104/−0.00027	0.00001	−11.6	−26.3/−6.98	0.00049	11.6	6.98/26.3	0.00049
	Female	−0.00039	−0.00075/−0.00026	0.00001	−9.11	−17.4/−6.01	0.00024	9.11	6.01/17.4	0.00024
***E. bicolor***	Male	−0.00090	−0.00209/0.00002	0.00049	−8.73	−18.0/2.13	0.00049	8.73	2.13/18.0	0.00024
	Female	−0.00110	−0.00251/−0.00043	0.00005	−10.7	−31.1/−5.30	0.00098	10.7	5.30/31.1	0.00098
***O. latipes***	Male	−0.00008	−0.00033/0.00012	0.02789	−2.23	−8.89/3.51	0.05225	2.95	0.34/8.89	0.00049
	Female	−0.00010	−0.00071/−0.00001	0.00012	−2.58	−20.1/−0.16	0.00024	2.58	0.16/20.1	0.00024
***P. reticulata***	Male	−0.00030	−0.00040/−0.00002	0.00001	−9.11	−10.9/−0.72	0.00024	9.11	0.72/10.9	0.00024
	Female	−0.00033	−0.00056/−0.00010	0.00001	−8.95	−15.4/−2.82	0.00049	8.95	2.82/15.4	0.00049
***F. gardneri***	Male	−0.00018	−0.00097/0.00024	0.07429	−4.10	−21.2/10.6	0.10547	6.66	0.14/21.2	0.00195
	Female	0.00020	−0.00009/0.00034	0.00075	6.52	−2.79/12.0	0.00488	6.52	2.44/12.0	0.00098
***P. scalare***	Male	−0.00068	−0.00253/0.00205	0.41237	−1.69	−12.3/11.6	0.37500	2.96	1.00/12.3	0.00195
	Female	−0.00027	−0.00216/0.00153	0.36028	−4.55	−14.3/11.1	0.36523	8.13	0.28/14.3	0.00098
***B. splendens***	Male	−0.00030	−0.00056/−0.00019	0.00001	−10.2	−22.9/−4.92	0.00098	10.2	4.92/22.9	0.00098
	Female	−0.00028	−0.00086/−0.00005	0.00011	−8.25	−27.3/−2.00	0.00012	8.25	2.00/27.3	0.00012

Abbreviations: L (Left), Max (maximum value), Min (minimum value), R (Right).

### Differences in habenular asymmetry does not relate to body size, habenular volume, or sex in most teleost species

**Figure 3:**
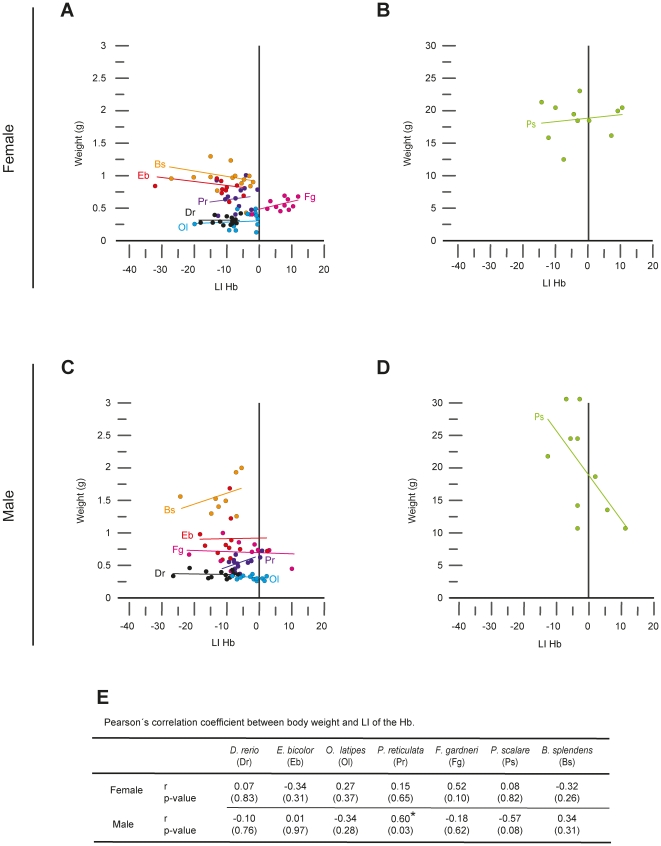
Relationship between body weight and habenular laterality Index. (A–D) Plots showing the relation between the weight of individual fish (in grams, g) and the score of habenular laterality index (LI) in different species of teleosts. Positive and negative scores of LI indicate right- and left- sided direction of habenular asymmetry, respectively. Data corresponding to different species have been presented in two rows, each representing a sex (female = top; male = bottom), and grouped into two columns according to the size of individuals (left = smaller fish; right = larger fish). Groups of dots sharing the same colour correspond to individuals of a single species, and the line of equivalent colour depicts the linear regression of that group. The abbreviation for each species is given on either left or right sides of the regression line, according to the code given in E. (E) Pearson's correlation coefficient (r) and p values (in parenthesis) for each species and sex. The asterisk indicates the presence of statistically significant correlation between body weight and habenular LI (p<0.05).

**Figure 4:**
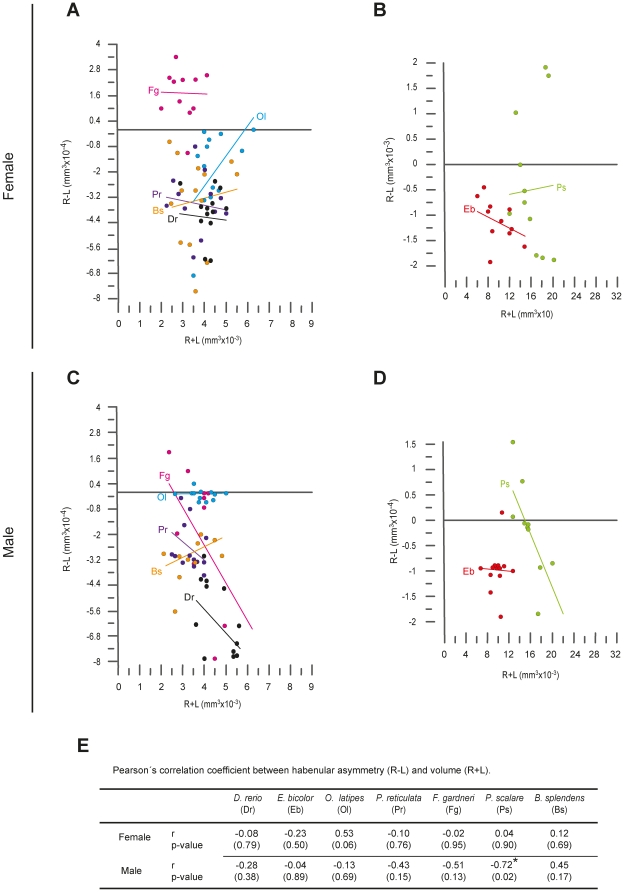
Relationship between habenular volume and laterality Index. (A–D) Plots showing the relation between habenular volume (rHb+lHb, in mm^3^×10^−3^) and asymmetry (rHb - lHb, in mm^3^×10^−4^) in different species of teleosts. Data corresponding to different species have been presented in two rows, each representing sex (female = top; male = bottom), and grouped into two columns according to the size of individuals (left = smaller fish; right = larger fish). Groups of dots sharing the same colour correspond to individuals of a single species, and the line of equivalent colour depicts the linear regression of that group. The abbreviation for each species is given on either left or right sides of the regression line, according to the code given in E. (E) Pearson's correlation coefficient (r) and p values (in parenthesis) for each species and sex. The asterisk indicates the presence of statistically significant correlation between habenular volume and asymmetry (p<0.05).

We next determined whether variations in the strength and laterality of habenular asymmetry among individuals of a single species were correlated with body size or habenular volume, or if they depended on sex. Pearson's correlation coefficient analysis revealed that habenular asymmetry (LI) was not correlated with body weight (Fig. 3) or standard length (data not shown; p>0.05) in all examined species, with the sole exception of P. reticulata, in which larger male individuals showed significantly less strength of habenular asymmetry compared to smaller male individuals (Fig. 3C - Pr; p = 0.03). Similarly, habenular asymmetry did not show significant correlation to total habenular volume with the sole exception of P. scalare. In male individuals of this species, a larger Hb was associated with left-sided asymmetry while a smaller Hb was associated with either symmetry or right-sided volumetric asymmetry (Fig. 4D - Ps; p = 0.02).

The presence of significant sex-specific differences in population level asymmetry of the Hb was also infrequent in teleosts, and only reached statistical significance in D. rerio and F. gardneri. In F. gardneri, differences appeared as a consequence of population level asymmetry being significant in females but absent in males (Fig. 2D and Table 2; p = 0.0064), with an additional pronounced dissimilarity in the scores of LI among sex (LI*_Fg_*_-male_ = −4.10; LI*_Fg_*_-female_ = 6.52) (Fig. 2E and Table 2; p = 0.0045). Importantly, in spite of the observed differences in habenular LI, the strength of asymmetry at the individual level was not significantly different among sex in F. gardneri (abs-LI*_Fg_*_-male_ = 6.66; abs-LI*_Fg_*_-female_ = 6.52) (Fig. 2F and Table 2; p = 0.62), or in all other species (Table 2). On the other hand, sex-specific differences observed in D. rerio were due to males having stronger left-sided population level asymmetry of the Hb than females (Figure 2D and Table 2; p = 0.035). Interestingly, the absolute volume of the left Hb was significantly larger in males than in females (Table 2; p = 0.01), while the volume of the right Hb did not show significant differences among sex (Table 2). Sex-specific differences in absolute volume of the left Hb were also significant in F. gardneri (males>females; p = 0.01), O. latipes (females>males; p = 0.01) and P. reticulata (females>males; p = 0.04) but these differences did not associate with significant sex-specific differences in habenular asymmetry (Fig. 2D and Table 2).

**Table 2:** Sex-specific differences in volume, asymmetry and laterality index of the habenulae in teleosts.

	Absolute Habenular Volume			Asymmetry [R-L]≠0	Laterality Index	
	Total (R+L)	Right (R)	Left (L)		Directional	Absolute
	*p*-Value	*p*-Value	*p*-Value	*p*-Value	*p*-Value	*p*-Value
***D. rerio***	0.029 [m]	0.15	0.01 [m]	0.035	0.17	0.25
***E. bicolor***	0.68	0.51	0.88	0.52	0.38	0.38
***O. latipes***	0.02 [f]	0.07	0.01 [f]	0.26	0.40	0.91
***P. reticulata***	0.07	0.11	0.04 [f]	0.12	0.35	0.35
***F. gardneri***	0.03 [m]	0.16	0.01 [m]	0.0064	0.0045	0.62
***P. scalare***	0.79	0.97	0.72	0.76	0.61	0.09
***B. splendens***	0.37	0.44	0.32	0.68	0.49	0.49

Abbreviations: male>female [m], female>male [f].

### Habenular asymmetry and its relation to social vs non-social behaviour

Previous studies in teleosts have reported a relation between population level behavioural lateralisation in a detour test and social behaviour [15]. We thus examined whether the frequency, strength or laterality of habenular volume related to “sociability” in the examined species. For this, we compared volumetric asymmetries of the Hb, at both individual and population levels, in pairs of species belonging to the same order, but in which one species was social and the other showed solitary/aggressive behaviour. Comparison across orders revealed no consistency in the relation between habenular asymmetry and social/aggressive behaviour. For example, D. rerio (social) and E. bicolor (solitary/aggressive) showed no differences in habenular asymmetry, in either males or females, at both individual and population levels (Table 3). In contrast, females of P. reticulata (social) and F. gardneri (solitary/aggressive) displayed statistically significant differences in habenular laterality at the population level, P. reticulata being lateralised to the left while F. gardneri to the right (Table 3, p = 0.001). On the other hand, males of P. scalare (social) and B. splendens (solitary/aggressive) showed significant differences in habenular asymmetry at both individual and population levels (Table 3; p = 0.015 and p = 0.002, respectively).

**Table 3:** Social vs Non-social differences in the laterality index of the habenulae in teleosts.

		Laterality Index	
		Directional	Absolute
	Sex	*p*-Value	*p*-Value
***D. rerio vs E. bicolor***	Male	0.087	0.087
	Female	0.073	0.073
***P. reticulata vs F. gardneri***	Male	0.300	0.690
	Female	0.001	0.063
***P. scalare vs B. splendens***	Male	0.002	0.015
	Female	0.042	0.440

### Segregation of left-right habenular efferents along dorso-ventral domains of the interpeduncular nucleus is largely conserved among teleosts

**Figure 5:**
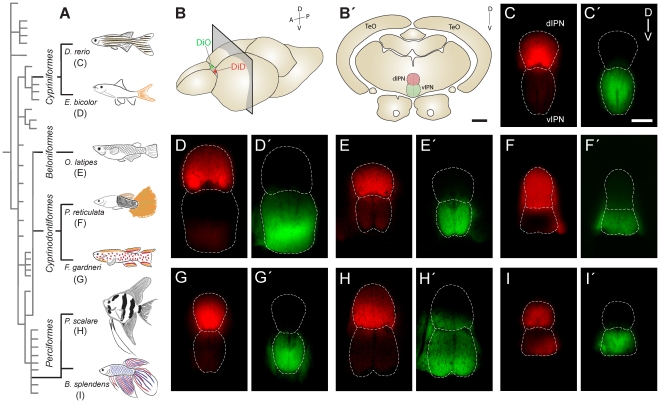
Comparative distribution of left and right habenular efferents in the interpeduncular nucleus of teleosts. (A) Drawings of adult male individuals belonging to different teleost species, placed in the context of a cladogram of the teleost lineage according to Nelson [28]. (B) Schematic representation of a teleost brain (e.g. D rerio), showing the procedure of differential dye labelling in left (DiD, red) and right (DiO, green) Hb, and the location and orientation of the histological sections shown in B′, and C–I. (B′) Schematic drawing of a coronal section at the level of the IPN in a teleost brain (e.g. D rerio), showing dorsal and ventral aspects of the IPN in red and green, respectively. (C–I) Confocal microscopy images of 100 µm-thick vibratome sections taken according to B′, with dorsal to the top. The boundaries of dorsal (dIPN) and ventral (vIPN) IPN domains have been depicted with dashed lines. Panels C to I correspond to efferents labelled after placing crystals of DiD in the left Hb. Panels C′ to I′ correspond to efferents labelled after placing crystals of DiO in the right Hb. Abbreviations: A (anterior), D (dorsal), P (posterior), TeO (Optic Tectum), V (ventral). Scale bars: 100 µm.

To determine whether left and right habenular efferents display selective target connectivity to dorsal and ventral domains of the IPN, we differentially labelled left and right habenular efferent projections by placing crystals of different carbocyanine dyes (DiO and DiD) in either the left or right Hb of fixed adult male brains (Fig. 5B). For F. gardneri, we also included female brains in the analysis as female individuals of this species showed right-sided directional asymmetry of the Hb (Fig. 2D). A comparative view of the obtained Hb-IPN connectivity patterns revealed a striking overall conservation in the segregation of left-right habenular efferents into dorsal and ventral domains of the IPN in all examined teleost species, as seen in coronal sections revealing the entire dorso-ventral extent of the IPN (Fig. 5B′). DiD carbocyanine crystals placed in the left Hb typically generated strong labelling of the dorsal aspect of the IPN (dIPN) with much less staining in the ventral IPN (vIPN) (Fig. 5C–I). Staining of the vIPN varied slightly among species, being very weak in D. rerio (Fig. 5C), E. bicolor (Fig. 5D), P reticulata (Fig. 5F), F. gardneri males (Fig. 5G), and P. scalare (Fig. 5H), and slightly more pronounced in O. latipes (Fig. 5E), B. splendens (Fig. 5I) and females of F gardneri (not shown). Despite these differences, staining was always notoriously more robust in the dIPN than in the vIPN (Fig. 5C–I). In contrast, DiO carbocyanine crystals placed in the right Hb induced strong and almost exclusive labelling of the vIPN, which was remarkably conserved among all species examined (Fig. 5C′–I′). In some species, such as P. scalare, labelling extended slightly into dorsal positions, likely encompassing the intermediate region of the IPN (Fig. 5H′).

## Discussion

To our knowledge, this is the first systematic inter-species comparative approach of structural asymmetries and efferent connectional properties of the Hb within a single vertebrate group. Among the species of teleosts examined, we found striking variability in habenular asymmetric morphology and in the frequency, strength and laterality of volumetric asymmetry of the Hb at the population level, which coexisted with an overall conserved laterotopic segregation of left and right habenular efferents in dorsal and ventral domains of the IPN, respectively. These findings reveal intrinsic plastic properties of the Hb, and suggest a fundamental role of its main efferent connectivity output within the teleost group. They also highlight the usefulness of comparative approaches in unveiling morpho-functional properties of distinct circuit designs.

### Evolutionary variation of habenular asymmetry and the role of phylogeny, behaviour and sex

This study revealed that the overall shape, rostro-caudal extension and cytoarchitectonic organisation of neuropil and cellular domains of the teleost Hb greatly differ among teleosts, a feature that is specially evident among species of different orders, and to a lesser extent within a single order. Variability in habenular cytoarchitecture was evident when testing the ability to distinguish the borders that separate dHb from vHb, which was unambiguous in both species of Cypriniformes (D. rerio and E. bicolor), partially clear in F. gardneri and P. scalare, and rather obscure in the remaining species. These findings are consistent with previous cytoarchitectonic reports in teleosts, most of which show the Hb at a very low resolution [10], [16], [17], [18], [19], [20], [21], [22], [23], [24], and contrast with the ease to distinguish the mammalian homolog of the teleost dHb and vHb, known as medial and lateral habenular nuclei, respectively [25], [26], [27]. Inter-species variability also extended to several aspects of the distribution of left and right habenular volumes. Left-right volumetric asymmetries of the Hb were present at the individual level in all species examined and thus represent a fundamental feature of the teleost group. However, the strength of habenular asymmetry at the individual level (as measured by the abs-LI) varied among species, being conspicuous in D. rerio, E. bicolor, B. splendens, P. reticulata and females of P. scalare, intermediate in F. gardneri, and rather weak in O. latipes and males of P. scalare. Furthermore, the laterality of habenular volume at the population level largely varied among species, being directed to either left (D. rerio, E. bicolor, B. splendens, P. reticulata and O. latipes) or right (females of F. gardneri), and even absent in some species (P. scalare and males of F. gardneri). Finally, variations in habenular asymmetry also extended to individuals within a single species. Altogether, the observed inter-species variability in habenular asymmetric morphology may reflect intrinsic plastic properties of the asymmetric circuit design involving the Hb of teleosts, which appears to promote structural flexibility depending on species- and sex-specific developmental, cognitive and/or behavioural pressures.

A possible source of inter-species variation is phylogenetic history. This is supported by the strong variability of habenular asymmetry among species of different vertebrate groups [3], and may be especially relevant for the teleost species examined in this study as they belong to different superorders (Ostariophysi, Euteleostei) and orders (Cypriniformes, Cyprinodontiformes, Beloniformes, Perciformes), and therefore share a long-history of independent evolution [28]. Indeed, we observed that inter-species variability in habenular asymmetric morphology was more pronounced across orders than within a single order. However, large variations were still observed among species of the same order, indicating that factors other than phylogeny play fundamental roles in the observed variations. Previous studies in the cichlid fish Geophagus brasiliensis have shown that individual differences in habenular asymmetry may correlate with growth [10]. However, we found no significant correlation between habenular asymmetry (LI) and standard length (or body weight) in the examined species, with the exception of a single species and sex, suggesting that growth plays a marginal role in the variability of habenular asymmetry. In addition, a comparative analysis of 16 species of teleosts, some of which were included in this study, revealed that behavioural lateralisation in a detour test relates to the “sociability” of the species. For example, social species show significant behavioural lateralisation at the population level while solitary/aggressive species only exhibit lateralisation at the individual level [15]. Other studies have associated aggressiveness with individual differences in behavioural lateralisation in some teleost species [29], [30]. In this study, we found that for some species and sex, the laterality of habenular asymmetry was significantly different between social and solitary/aggressive species of the same order. It has been suggested that consistency in the laterality of behavioural lateralisation might help coordinate behaviour among individuals [15], and that differences in personality (e.g. aggressiveness) can influence cerebral lateralisation [29] and be maintained in phylogeny through frequency-dependent selection [31]. Our results support the notion that the social vs solitary/aggressive nature of the species may be a source of evolutionary variation in brain asymmetry, although further studies will be required to understand the significance of these observations in the context of habenular asymmetry.

Sex is a known source of inter-species variation in brain asymmetry across vertebrates [32]. Studies in amphibians and birds have shown that habenular asymmetry can be influenced by steroid hormones and thus be a subject of variability among sex. For example, left-sided asymmetry of the dorsal Hb in Rana esculenta is more pronounced in females than males, especially during the reproductive season [33] whereas in chicks the medial Hb shows right-sided volumetric asymmetry only in males [9]. We found that sex-specific differences in volumetric asymmetries of the Hb are infrequent in teleosts, and thus are not a primary source of inter-species variations. This is consistent with previous reports showing equal volumetric asymmetries of the Hb in males and females of cichlid fish (Geophagus brasiliensis and Amatitlania nigrofasciata). However, in those species there was a sex-specific correlation between the direction of habenular asymmetry and the direction of behavioural lateralisation in a detour test [10], [16]. Importantly, our analysis showed that the source of sex-specific differences in habenular asymmetry varied among species, as described for chick and amphibians. In F. gardneri, sex-specific differences were the result of asymmetry at the population level developing exclusively in females but not in males, although the strength of asymmetry at the individual level was similar in both sexes. Interestingly, a study in a non-social aggressive teleost (the convict cichlid Archocentrus nigrofasciatus) showed a link between sex-specific differences in behavioural lateralisation in a detour test, and aggressiveness [29]. As males of F. gardneri appear more aggressive than females [34], it would be interesting to explore whether aggressiveness in this species plays a role in the observed sex-specific differences in habenular asymmetry. On the other hand, sex-specific differences in D. rerio were a consequence of males showing a stronger population level asymmetry than females. Such sex-specific differences were associated with an imbalanced growth of the left Hb in males compared to females, although the meaning of this observation remains to be solved.

### Developmental plasticity as a source of evolutionary variation in habenular asymmetry

Recent work in zebrafish (D. rerio) indicates that asymmetric morphogenesis of the Hb depends on the tight regulation of neurogenic periods responsible for generating the two main neuronal subtypes of the Hb, known as lateral and medial [35], [36]. An early onset of neurogenesis seems responsible for generating a larger proportion of lateral compared to medial neurons in the left Hb, while a delay in this process explains the larger proportion of medial vs lateral neurons in the right Hb [36]. The epithalamic parapineal organ (PpO), a photosensitive structure that forms at the midline and migrates to the left side to connect the left Hb [37], [38], is proposed to play a key role in inducing the anticipated timing of neurogenesis in the left Hb and thus establishing the asymmetric ratio of lateral vs medial neurons [39]. Indeed, genetic and physical ablation of the PpO in zebrafish results in a symmetric proportion of lateral vs medial neurons in each side of the Hb [38], [40], [41]. Importantly, as lateral and medial habenular neurons display target selectivity to dorsal and ventral domains of the IPN, their asymmetric enrichment in left and right sides results in dorso-ventral segregation of left-right habenular efferents in the IPN, respectively. The present study revealed that in all species examined, efferent connectivity from left and right Hb preferentially innervate dorsal and ventral domains of the IPN, respectively, therefore indicating that asymmetry in the ratio of lateral vs medial habenular neurons is a conserved trait among teleosts. Such conservation contrasts with the large degree of variation of habenular asymmetric morphology, and suggests that the ontogenic mechanisms responsible for generating morphological/volumetric asymmetries of the Hb are uncoupled from those that generate asymmetry in the ratio of lateral vs medial habenular neurons. Thus, what are the ontogenic mechanisms underlying the laterality of habenular volume?

In addition to its proposed role in regulating the timing of neurogenesis in the left Hb [39], the PpO also seems to be crucial for the modulation of asymmetric morphogenesis. Indeed, the elaboration of structural asymmetries of the Hb in zebrafish larvae, expressed as differences in the content of neuropil, largely depend on the proper left-sided migration of the PpO. Indeed, parapineal axons terminate in those neuropil regions of the left Hb that show the most conspicuous morphological asymmetries [13], [38], and situations in which the PpO has been removed associate with development of an overall symmetric habenular neuropil [38], [40], [41]. It thus appears that PpO-Hb interactions play a fundamental role in shaping asymmetric morphogenesis of the Hb, a proposal that is supported by a recent comparison of habenular asymmetric development between D. rerio and O. latipes [13]. This study showed that conspicuous heterotopic transformations in habenular asymmetric morphology appeared in the evolution of both species, and that these changes associate with a heterochronic shift in the onset of connectivity between the PpO and left Hb, and with differences in the topology of the habenular domain that receives parapineal efferent connectivity [13]. We thus propose that the PpO exerts a local influence on the left Hb that serves to shape the organisation of cellular and neuropil domains during habenular morphogenesis, and that this feature may vary among species according to changes in the timing and/or topology of PpO-Hb interactions. The proposed local influence of PpO-Hb interaction in morphogenesis is in contrast to the more global influence of the PpO in neurogenesis, which appears to propagate from the region of influence to a broader region of the left Hb. Such proposed autonomy and uncoupling in the influence of the PpO in morphogenesis and neurogenesis is consistent with two relevant findings of this study. First, a dissociation in the degree of inter-species variations in two main aspects of habenular asymmetry, named morphology/volume (high variation) and ratio of lateral-vs-medial neurons (low variation, as expressed by the conserved laterotopic pattern of Hb-IPN connectivity). Second, the association of large volumetric asymmetry of the Hb in species where the PpO has a broader spatial influence during development, as reflected by the area covered by efferents of the PpO. For example, the region of the left Hb covered by efferents of the PpO is extended in D. rerio but notoriously localised in O. latipes [13], and this inter-species difference associates to a stronger volumetric asymmetry of the Hb in D. rerio (LI*_Dr_*_-female_ = −9.1; LI*_Dr_*_-male_ = −11.6) compared to O. latipes (LI*_Ol_*_-female_ = −2.6; LI*_Ol_*_-male_ = −2.2). Importantly, our proposal can be experimentally tested in the future through combining comparative analyses of parapineal connectivity among teleost species, and experimental manipulation of parapineal efferent connectivity during ontogeny.

### Laterotopic conservation of habenular efferents and the developmental modular organisation of epithalamic asymmetry

A fundamental finding of this work is that, in spite of the notorious inter-species variations in the presence, strength and laterality of habenular asymmetric morphology, the overall connectivity of left-right habenular efferents shows a conserved pattern of segregation along dorso-ventral domains of the IPN. This observation implies that the ontogenic mechanisms responsible for organising left and right habenular efferents within the IPN are much less plastic than those involved in habenular asymmetric morphogenesis, perhaps due to the pivotal role that this efferent pattern plays in circuit physiology. Indeed, the circuit involving the Hb plays a pivotal and conserved function in the control of dopaminergic and serotonergic systems among vertebrates [35], [42], and genetic inhibition of the asymmetric circuit that primarily involves the left Hb and dIPN in zebrafish leads to a defective enhancement of fear responses and anxiety [43], [44] while the complementary circuit involving the right Hb and vIPN is proposed to play an opposite regulatory role in these behaviours [45]. A possible evolutionary explanation for the co-existence of high variability in habenular asymmetric morphology with overall conservation of Hb-IPN connectivity comes from the proposed developmental modular organisation of epithalamic asymmetry, whose main outcome is the laterotopic segregation of habenular efferents in the IPN [39]. In this model, the ontogeny of epithalamic asymmetry organises into a sequence of interconnected developmental modules, in which the output of a module functions as the input in a subsequent module, within which it modulates the activity of some internal process. One main prediction of developmental modular organisation is that individual modules may change in a quasi-independent manner during ontogeny and phylogeny [46], [47], [48]. For instance, it is possible that inter-species variations in the input of the “habenular asymmetric morphogenesis module”, expressed as differences in the timing and/or topology of PpO-Hb interactions, generates distinct patterns of habenular asymmetric morphogenesis without largely affecting the overall asymmetric organisation of neurogenesis, and consequently the output asymmetric organisation of lateral vs medial neurons in the Hb. Importantly, the proposed dissociation in the PpO-dependent control of neurogenesis and morphogenesis in the context of the developmental modular organisation opens the possibility of having a Hb with volumetric asymmetries but being symmetric in the ratio of lateral vs medial neurons. Although this example has not yet been found in nature, the large diversity of teleosts species, each having remarkably derived phylogenetic histories, suggest that we just need to await this possibility.

## Materials and Methods

### Animals

Male and female individuals of seven species of teleosts were used. The criteria for species selection included: similar body size at adult stages, species of four different orders within Ostariophysi and Euteleostei groups, and distinct social behaviour (social vs solitary/aggressive). The chosen species were from Ostariophysi: Danio rerio and Epalzeorhynchos bicolor (Order Cypriniformes); and Euteleostei: Oryzias latipes (Order Beloniformes); Poecilia reticulata and Fundulopanchax gardneri (Order Cyprinodontiformes); Pterophyllum scalare and Betta splendens (Order Perciformes). D. rerio, O. latipes, P. reticulata and P. scalare show social behaviour while E. bicolor, F. gardneri and B. splendens are aggressive [15], [34]. B. splendens and E. bicolor were obtained from local pet stores while other species were raised at the fish facility of the Laboratory of Experimental Ontogeny (LEO), Faculty of Medicine, Universidad de Chile. The Ethics Commission of the Faculty of Medicine, University of Chile, under the Bioethics Protocol N° CBA# 0293 FMUCH, approved all the procedures performed in this study.

### Histology

Animals were anaesthetised with 5% tricaine, the weight and standard length of every specimen determined, and the heads removed and fixed by immersion in 4% paraformaldehyde (PFA) in 0.1 M saline phosphate buffer, pH 7.4 (PBS). The head tissue was decalcified using 5% EDTA in 0.1 M PBS, dehydrated through an increasing series of ethanol, and embedded in paraffin. Serial coronal sections (10 µm thick) were obtained using a microtome (Leitz, Wetzlar) and collected on gelatinised slides and subsequently stained with cresyl-violet (Nissl staining) [49] (Fig. 1B). Microphotographs of all sections containing the Hb were obtained using an upright Nikon Eclipse 80i microscope coupled with a digital camera (Nikon) and the NIS Elements Software.

### Image processing, 3D reconstructions and statistical analysis

Digital microphotographs were used to create z stacks using Image J Software (W.S. Rasband, U.S. National Institutes of Health; htpp://rsb.info.nih.gov/ij/, 1997–2005). Images corresponding to a series of coronal sections were registered, the borders of the Hb manually segmented, and three-dimensional volumetric reconstructions generated to measure habenular volume using Volocity software (Perkin Elmer). For each species and sex, total values in cubic microns corresponding to the volume of the right Hb (rHb) and left Hb (lHb) were used to obtain the x value (x = [rHb-lHb]), which was then used for statistical analysis of asymmetry and of sex-specific and behaviour-specific differences. Parametrical statistical approaches were used when data showed a normal distribution (as determined by Shapiro Wilk), while non-parametric tests were used in all other cases (Origin Pro8 Software). Either two tailed one-sample t test (parametric) or one sample Wilcoxon signed rank test (non-parametric) were used to determine the presence of asymmetry within a population, testing if the mean or median of x was different from 0, respectively. Either two tailed two-sample t test (parametric) or Mann-Whitney test (non-parametric) were used to determine differences between two populations of different sex (males vs females) or social behaviour (social vs solitary/aggressive). The laterality index (LI) was determined to examine the direction and intensity of population level asymmetry of the Hb, using the following formula: LI = 100×[rHb-lHb]/[rHb+lHb]. The intensity of habenular asymmetry at the individual level was obtained using absolute values of LI (abs-LI). Correlations between habenular LI and body weight and standard length, and between asymmetry (rHb-lHb) and habenular volume (rHb+lHb) were determined using the Pearson's Correlation Coefficient. The number of animals used for statistical analysis were as follows: D. rerio (n = 13 females and n = 12 males), E. bicolor (n = 11 females and n = 13 males), O. latipes (n = 13 females and n = 12 males), P. reticulata (n = 12 females and n = 13 males), F. gardneri (n = 11 females and n = 10 males), P. scalare (n = 11 females and n = 10 males), and B. splendens (n = 14 females and n = 11 males).

### Neurotracing with carbocyanine dyes

Male adult fish were anaesthetised with 5% tricaine and their heads removed and fixated with 4%PFA in 0.1 M PBS for 1 h. Brains were dissected and immersed again in 4% PFA for several days at 4°C and then placed in a sylgard coated dish, in a dorsal view, under the dissecting microscope. Dye labelling was performed through careful placement of crystals of DiD or DiO (Life Technologies Corporation) on the surface of either the left or right Hb, respectively (Fig. 5B). To avoid transmission of dye between both sides of the Hb, the habenular commissure was cut with a micro blade, and a piece of brain tissue introduced between both sides of the Hb. Brains were then incubated at 37°C for 2.5 days (D. rerio, P. reticulata), 5 days (B. splendens, E. bicolor) or 6 days (P. scalare, F gardneri, O. latipes). After incubation, coronal sections of the IPN (100 µm thick) (Fig. 5B′) were obtained using a vibratome (Leica), collected on gelatinised slides, and digital images taken using an Olympus spectral confocal microscope (Fluoview FV1000).

## References

[1] Bianco IH, Wilson SW (2009). The habenular nuclei: a conserved asymmetric relay station in the vertebrate brain.. Philos Trans R Soc Lond B Biol Sci.

[2] Hikosaka O (2010). The habenula: from stress evasion to value-based decision-making.. Nat Rev Neurosci.

[3] Concha ML, Wilson SW (2001). Asymmetry in the epithalamus of vertebrates.. J Anat.

[4] Nieuwenhuys R (1977). The brain of the lamprey in a comparative perspective.. Annals of the New York Academy of Sciences.

[5] Guglielmotti V, Cristino L (2006). The interplay between the pineal complex and the habenular nuclei in lower vertebrates in the context of the evolution of cerebral asymmetry.. Brain Res Bull.

[6] Engbretson GA, Reiner A, Brecha N (1981). Habenular asymmetry and the central connections of the parietal eye of the lizard.. Journal of Comparative Neurology.

[7] Wree A, Zilles K, Schleicher A (1981). Growth of fresh volumes and spontaneous cell death in the nuclei habenulae of albino rats during ontogenesis.. Anatatomy and Embryology.

[8] Zilles K, Schleicher A, Wingert F (1976). Quantitative analyse des wachstums der frischvolumina limberscherkerngebiete im diencephalon und mesencephalon einer ontogenetischen reihe von albinomäusen. 1. nucleus habenulare.. J Hirnforsch.

[9] Gurusinghe CJ, Ehrlich D (1985). Sex-dependent structural asymmetry of the medial habenular nucleus of the chicken brain.. Cell and Tissue Research.

[10] Reddon AR, Gutierrez-Ibanez C, Wylie DR, Hurd PL (2009). The relationship between growth, brain asymmetry and behavioural lateralization in a cichlid fish.. Behav Brain Res.

[11] Kuan YS, Gamse JT, Schreiber AM, Halpern ME (2007). Selective asymmetry in a conserved forebrain to midbrain projection.. J Exp Zool B Mol Dev Evol.

[12] Aizawa H, Bianco IH, Hamaoka T, Miyashita T, Uemura O (2005). Laterotopic representation of left-right information onto the dorso-ventral axis of a zebrafish midbrain target nucleus.. Curr Biol.

[13] Signore IA, Guerrero N, Loosli F, Colombo A, Villalon A (2009). Zebrafish and medaka: model organisms for a comparative developmental approach of brain asymmetry.. Philos Trans R Soc Lond B Biol Sci.

[14] Amo R, Aizawa H, Takahoko M, Kobayashi M, Takahashi R (2010). Identification of the zebrafish ventral habenula as a homolog of the mammalian lateral habenula.. J Neurosci.

[15] Bisazza A, Cantalupo C, Capocchiano M, Vallortigara G (2000). Population lateralisation and social behaviour: a study with 16 species of fish.. Laterality.

[16] Gutierrez-Ibanez C, Reddon AR, Kreuzer MB, Wylie DR, Hurd PL (2011). Variation in asymmetry of the habenular nucleus correlates with behavioural asymmetry in a cichlid fish.. Behav Brain Res.

[17] Schnitzlein HN (1962). The habenula and the dorsal thalamus of some teleosts.. J Comp Neurol.

[18] Steyn W, Webb M (1960). The pineal complex in the fish Labeo umbratus.. The Anatomical record.

[19] Braitenberg V, Kemali M (1970). Exceptions to bilateral symmetry in the epithalamus of lower vertebrates.. Journal of Comparative Neurology.

[20] Gómez-Segade P, Anadón R (1988). Specialization in the diencephalon of advanced teleosts.. Journal of morphology.

[21] Cerdá-Reverter J, Zanuy S, Muñoz-Cueto J (2001). Cytoarchitectonic study of the brain of a perciform species, the sea bass (Dicentrarchus labrax). II. The diencephalon.. Journal of morphology.

[22] Shanklin WM (1935). On diencephalic and mesencephalic nuclei and fibre paths in the brains of three deep sea fish.. Phil tr Roy Soc Lon series B.

[23] Holmgren N (1920). Zur Anatomie und Histologie des Vorder- und Zwischenhirns der Knochenfische. Hauptsächlich nach Untersuchungen an *Osmerus eperlanus*.. Acta Zoologica.

[24] Miller R (1940). The diencephalic cell masses of the teleost, *Corydora paliatus*.. The Journal of Comparative Neurology.

[25] Jones EG (2006). The epithalamus. The Thalamus. Second ed.

[26] Andres KH, von During M, Veh RW (1999). Subnuclear organization of the rat habenular complexes.. J Comp Neurol.

[27] Diaz E, Bravo D, Rojas X, Concha ML (2011). Morphologic and immunohistochemical organization of the human habenular complex.. J Comp Neurol.

[28] Nelson JS (2006). Fishes of the world.

[29] Reddon AR, Hurd PL (2008). Aggression, sex and individual differences in cerebral lateralization in a cichlid fish.. Biol Lett.

[30] Clotfelter ED, Kuperberg ES (2007). Cerebral lateralization and its relationship to phylogeny and aggression in anabantoid fishes.. Brain Behav Evol.

[31] Wolf M, van Doorn GS, Leimar O, Weissing FJ (2007). Life-history trade-offs favour the evolution of animal personalities.. Nature.

[32] Bianki VL, Filippova EB (2001). Sex differences in lateralization in the animal brain.

[33] Kemali M, Guglielmotti V, Fiorino L (1990). The asymmetry of the habenular nuclei of female and male frogs in spring and in winter.. Brain Research.

[34] Ewing AW (1975). Studies on the behaviour of Cyprinodont fish. II. The evolution of aggressive behaviour in Old World rivulins.. Behaviour.

[35] Aizawa H, Amo R, Okamoto H (2011). Phylogeny and ontogeny of the habenular structure.. Front Neurosci.

[36] Aizawa H, Goto M, Sato T, Okamoto H (2007). Temporally regulated asymmetric neurogenesis causes left-right difference in the zebrafish habenular structures.. Dev Cell.

[37] Concha ML, Burdine RD, Russell C, Schier AF, Wilson SW (2000). A nodal signaling pathway regulates the laterality of neuroanatomical asymmetries in the zebrafish forebrain.. Neuron.

[38] Concha ML, Russell C, Regan JC, Tawk M, Sidi S (2003). Local tissue interactions across the dorsal midline of the forebrain establish CNS laterality.. Neuron.

[39] Concha ML, Signore IA, Colombo A (2009). Mechanisms of directional asymmetry in the zebrafish epithalamus.. Semin Cell Dev Biol.

[40] Bianco IH, Carl M, Russell C, Clarke JD, Wilson SW (2008). Brain asymmetry is encoded at the level of axon terminal morphology.. Neural Develop.

[41] Gamse JT, Thisse C, Thisse B, Halpern ME (2003). The parapineal mediates left-right asymmetry in the zebrafish diencephalon.. Development.

[42] Stephenson-Jones M, Floros O, Robertson B, Grillner S (2011). Evolutionary conservation of the habenular nuclei and their circuitry controlling the dopamine and 5-hydroxytryptophan (5-HT) systems.. Proc Natl Acad Sci U S A.

[43] Agetsuma M, Aizawa H, Aoki T, Nakayama R, Takahoko M (2010). The habenula is crucial for experience-dependent modification of fear responses in zebrafish.. Nat Neurosci.

[44] Lee A, Mathuru AS, Teh C, Kibat C, Korzh V (2010). The habenula prevents helpless behavior in larval zebrafish.. Curr Biol.

[45] Okamoto H, Agetsuma M, Aizawa H (2011). Genetic dissection of the zebrafish habenula, a possible switching board for selection of behavioral strategy to cope with fear and anxiety.. Dev Neurobiol.

[46] Wagner GP, Pavlicev M, Cheverud JM (2007). The road to modularity.. Nat Rev Genet.

[47] Winther RG (2001). Varieties of modules: kinds, levels, origins, and behaviors.. J Exp Zool.

[48] Bolker JA (2000). Modularity in development and why it matters to Evo-Devo.. Amer Zool.

[49] Nissl F (1892). Uber die Veranderungen der Gangliezellen am Facialisken des Kaninchens nach Ausreissung der Nerven.. Allgem Z f Psychiat.

